# Endoscopic landmarks corresponding to anatomical landmarks for esophageal subsite classification

**DOI:** 10.1002/deo2.273

**Published:** 2023-07-17

**Authors:** Ryu Ishihara, Yasuhiro Tani, Yuki Okubo, Yuya Asada, Tomoya Ueda, Daiki Kitagawa, Takehiro Ninomiya, Atsuko Tamashiro, Shunsuke Yoshii, Satoki Shichijo, Takashi Kanesaka, Sachiko Yamamoto, Yoji Takeuchi, Koji Higashino, Noriya Uedo, Tomoki Michida

**Affiliations:** ^1^ Department of Gastrointestinal Oncology Osaka International Cancer Institute Osaka Japan; ^2^ Department of Gastroenterology and Hepatology Gunma University Graduate School of Medicine Gunma Japan

**Keywords:** classification, endoscopy, esophagus, landmark, pharynx

## Abstract

**Objectives:**

Individual treatment strategies for esophageal cancer have been investigated based on the anatomical subsite classification. Accurate subsite classification based on these anatomical landmarks is thus important. We investigated the suitability of the existing endoscopic classification and explored alternative landmarks for esophageal subsite classification.

**Methods:**

Patients who received endoscopic ultrasonography (and computed tomography scans for surveillance of esophageal cancer treatment or esophageal submucosal tumors were included. Distances between anatomical landmarks, including the inferior cricoid cartilage border, superior border of the sternum, and tracheal bifurcation, were measured using a combination of endoscopic ultrasonography, computed tomography, and other information.

**Results:**

The mean (standard deviation) distances from the superior incisor dentition to the pharynx–esophagus, cervical–upper thoracic esophagus, and upper–middle thoracic esophagus boundaries were 16.9 (1.7), 21.7 (1.9), and 29.0 (1.9) cm, respectively. However, variances in the differences between the mean and individual distances were large (2.8, 3.4, and 3.7, respectively), mainly because of differences in body height. However, variances in the differences between individual distances and novel endoscopic landmarks, including the lower end of the pyriform sinus and lower end of compression of the left main bronchus, were lower (1.7, 1.2, and 0.6, respectively).

**Conclusions:**

Existing indicators of esophageal subsite boundaries were not consistent with anatomical boundaries. Modification of the distance from the superior incisor dentition based on average distances from anatomical landmarks or the use of alternative endoscopic landmarks is recommended to provide more suitable anatomical boundaries.

## INTRODUCTION

Esophageal cancer is the seventh most common cancer and the sixth most common cause of cancer‐related mortality worldwide.^1^ Squamous cell carcinoma is the predominant type of esophageal cancer in Asia.^2^ Endoscopic resection (is a curative treatment for esophageal cancers with negligible risk of metastasis,^3^ while additional treatment after endoscopic resection is recommended for cancers with a substantial risk of metastasis based on a pathological assessment of resected specimens.^4^, ^5^

Esophagectomy and chemoradiotherapy are the leading options for adjuvant therapy for esophageal squamous cell carcinoma after endoscopic resection.^4^, ^5^ The extent of lymphadenectomy during esophagectomy and the radiation field in chemoradiotherapy are determined based on the subsite of the original esophageal squamous cell carcinoma, such as the cervical esophagus, upper thoracic esophagus, middle thoracic esophagus, or lower thoracic esophagus.^5^, ^6^, ^7^, ^8^ The anatomical landmarks of the boundaries were as follows: the pharynx/cervical esophagus boundary was defined as the inferior border of the cricoid cartilage; the cervical/upper thoracic esophagus boundary was the superior border of the sternum; the upper/middle thoracic esophagus boundary was the tracheal bifurcation; and the middle/lower thoracic esophagus boundary was the midpoint of the tracheal bifurcation and esophagogastric junction.^9^ In addition, individual treatment strategies for esophageal cancers have been investigated according to the specific anatomical subsite classification,^10^, ^11^, ^12^ thus highlighting the importance of accurate subsite classification based on these anatomical landmarks.

The definition of esophageal subsite based on endoscopic findings is not described in the Japanese Classification of Esophageal Cancer^9^ or other documents. The definitions of anatomical landmarks, such as the inferior border of the cricoid cartilage, the superior border of the sternum, and tracheal bifurcation, cannot be observed by endoscopy. We can observe compression of the left main bronchus which has a 3–4 cm length and we don't know which part of the compression corresponds to tracheal bifurcation.

We, therefore, used distance from the superior incisor dentition (e.g., upper thoracic esophagus 18–24 cm from the superior incisor dentition) as the endoscopic landmark for esophageal subsites in multicenter trials^7^, ^13^ because of a lack of other adequate landmarks. However, this endoscopic landmark is not based on any data and its adequacy has not yet been evaluated. The aim of this study was to investigate the appropriateness of the existing classification and to explore alternative landmarks for esophageal subsite classification.

## METHODS

### Patients

The inclusion criteria for this study were patients who received endoscopic ultrasonography (EUS) and computed tomography (CT) scans for surveillance of esophageal cancer treatment or esophageal submucosal tumors. Patients were excluded if they had a scar in the left‐to‐anterior wall of the esophagus around 30 cm from the superior incisor dentition (inability to assess compression of left main bronchus), a scar in the cervical esophagus (inability to assess palisade vessels), or surgical resection of the pharynx or esophagus. Identification of indicators (vocal cord, lower end of the pyriform sinus, lower end of the palisade vessels, lower end of compression of the left main bronchus, azygos vein arch, and flexure of the subclavian artery) and documentation of the length from the superior incisor dentition was conducted on‐site during endoscopy. This was a retrospective analysis of data collected between January 2022 and February 2023. The need for written informed consent for study participation was waived because all participants had the opportunity to decline to participate using an opt‐out option on our hospital website.

### Identification and measurement of various indicators

Anatomical landmarks of the pharynx–cervical esophagus, cervical–upper thoracic esophagus, and upper–middle thoracic esophagus were defined as the inferior border of the cricoid cartilage, superior border of the sternum, and tracheal bifurcation, respectively, according to the Japanese Classification of Esophageal Cancer.^9^

**FIGURE 1:**
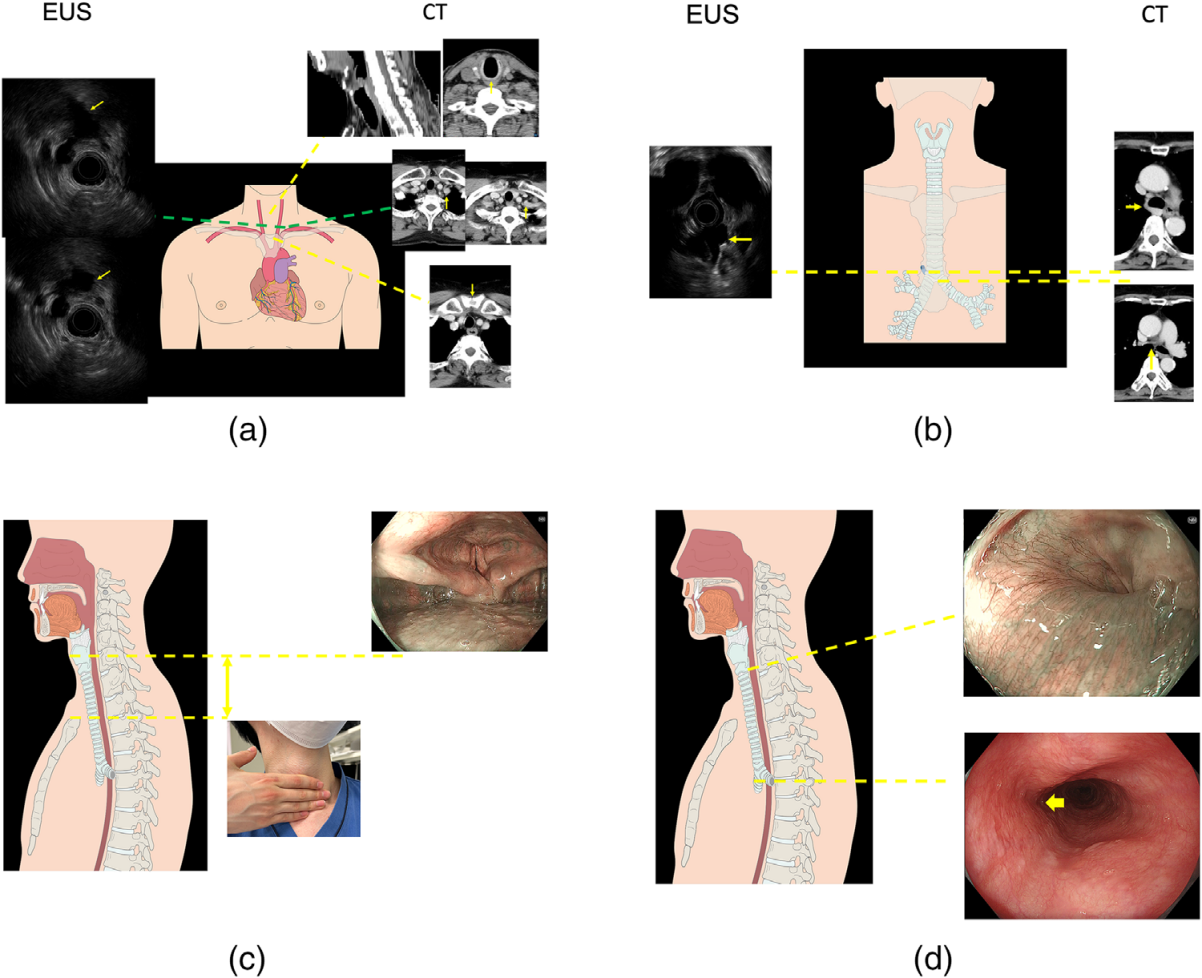
Measurement of indicators of esophageal subsite classification. (a) Distance from superior incisor dentition to flexure of subclavian artery measured by endoscopic ultrasonography (EUS). Distances between the inferior border of the cricoid cartilage and flexure of the subclavian artery ,and between flexure of the subclavian artery and superior border of the sternum measured by computed tomography (CT) scan. (b) Distance from superior incisor dentition to azygos vein arch recorded during the examination. Distance between tracheal bifurcation and azygos vein arch measured by CT scan. (c) Distances between superior incisor dentition and vocal cords, and between laryngeal prominence and superior border of sternum were measured. (d) Distances from the superior incisor dentition to the lower end of palisade vessels in the cervical esophagus, and the lower end of compression of the left main bronchus were measured.

Surveillance EUS was conducted for lymph node metastasis or esophageal submucosal tumors and landmarks including the splenic vein, celiac artery, left atrium, azygos vein, subclavian artery, and common carotid artery were identified (Figure 1a,b). We identified the splenic vein followed by the celiac artery because lymph nodes around the celiac artery and left gastric artery are common sites of esophageal cancer metastasis. The distances from the superior incisor dentition to the azygos vein arch and andflexure of the subclavian artery were recorded during the examination.

The distances between the inferior border of the cricoid cartilage and flexure of the subclavian artery, flexure of the subclavian artery and superior border of the sternum, and the tracheal bifurcation and azygos vein arch were measured on CT scans (Figure 1a,b).

The distance between the laryngeal prominence and the superior border of the sternum was measured by finger‐breadth measurement during a physical examination by a single endoscopist (Ryu Ishihara; Figure 1c).

Patients with esophageal cancer usually underwent meticulous conventional endoscopic observation of the pharyngeal area, and the distances from the superior incisor dentition to the vocal cords (Figure 1c), the lower end of the pyriform sinus (Figure 1d), the lower end of the palisade vessels in the cervical esophagus, and lower end of compression of the left main bronchus (Figure 1d)^14^ were measured.

### Measurement of anatomical landmarks

**FIGURE 2:**
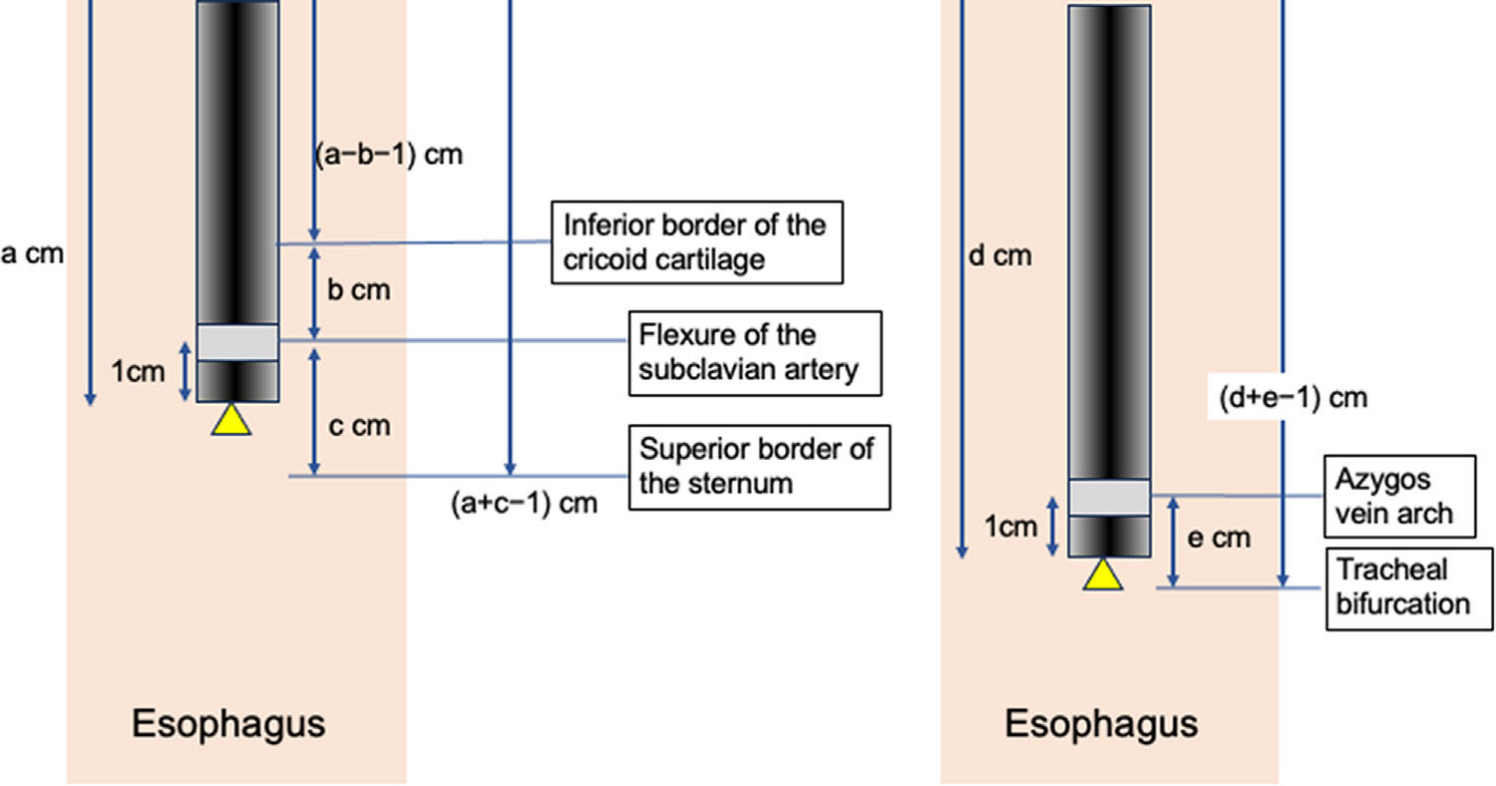
Measurement of anatomical landmarks.

The boundary of the pharynx and cervical esophagus was calculated by the distances between the superior incisor dentition and flexure of the subclavian artery (a), and between the inferior border of the cricoid cartilage and flexure of the subclavian artery (b), adjusted by the distance between the front edge of EUS and the EUS probe (1 cm; Figure 2). Therefore, the boundary of the pharynx and cervical esophagus = (a−b−1) cm from the superior incisor dentition.

The boundary of the cervical and upper thoracic esophagus was calculated by the distances between the superior incisor dentition and flexure of the subclavian artery (a) and between the flexure of the subclavian artery and superior border of the sternum (c), adjusted by the distance between the front edge of EUS and the EUS probe (1 cm; Figure 2). The boundary of the cervical and upper thoracic esophagus = (a+c−1) cm from the superior incisor dentition.

The boundary of the upper and middle thoracic esophagus was calculated by the distances between the superior incisor dentition and azygos vein arch (d) and between the azygos vein arch and tracheal bifurcation (e), adjusted by the distance between the front edge of EUS and the EUS probe (1 cm; Figure 2). The boundary of the upper and middle thoracic esophagus = (d+e−1) cm from the superior incisor dentition.

### Definition of endoscopic landmarks

In this study, the endoscopic landmarks of the pharynx–cervical esophagus and upper–middle thoracic esophagus boundaries were defined as the lower end of the pyriform sinus and lower end of compression of the left main bronchus, respectively (Figure 1d). The endoscopic landmark of the cervical–upper thoracic esophagus boundary was defined as the additional value of the distances from the superior incisor dentition to the vocal cords and the distance from the laryngeal prominence and superior border of the sternum (Figure 1c). The cervical–upper thoracic esophagus definition was based on the fact that the vocal cords and laryngeal prominence are adjacent to each other.

### Statistical analysis

The distance between the superior incisor dentition and each boundary was expressed as mean (± standard deviation; SD). The relationships between the distances of the boundaries from the superior incisor dentition and body height, body weight, and age were investigated by multiple linear regression analyses. The parameters were assessed by calculating the coefficient of determination (R^2^). R^2^ ranged from 0–1 and measured how well the statistical models predicted the distance of the boundary from the superior incisor dentition, with a higher value indicating a better prediction. Variances between groups were analyzed by the F‐test. For all analyses, a two‐sided *p‐*value < 0.05 was considered statistically significant. Statistical analyses were carried out using JMP version 16.2.0 (SAS Institute).

## RESULTS

### Factors associated with locations of boundaries

**FIGURE 3:**
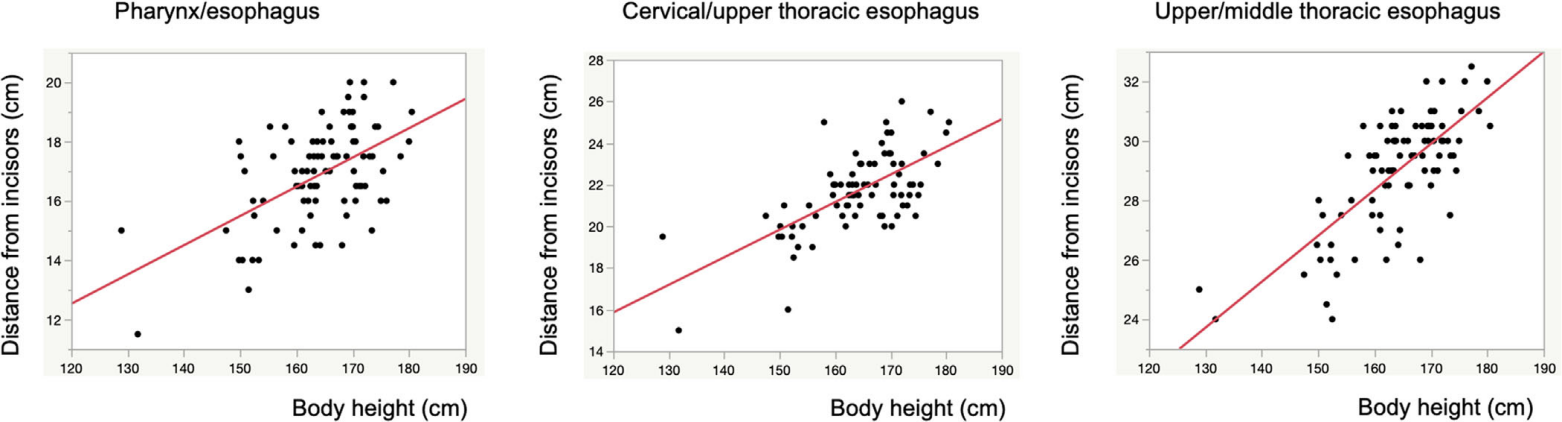
Associations between body height and distances from superior incisor dentition to pharynx and cervical esophagus, cervical and upper thoracic esophagus, and upper and middle thoracic esophagus boundaries.

Ninety‐four patients between January 2022 and February 2023 fulfilled the inclusion criteria. Seven patients were excluded because of scars in the cervical esophagus or left‐to‐anterior wall of the esophagus around 30 cm from superior incisor dentition, and 87 patients, therefore, fulfilled all the criteria. Boundary distances were measured based on physical examination, conventional endoscopy, EUS, and CT data. The mean (SD) distances of the pharynx–cervical esophagus, cervical–upper thoracic esophagus, and upper–middle thoracic esophagus boundaries were 16.9 (1.7), 21.7 (1.9), and 29.0 (1.9) cm, respectively. According to multiple linear regression analyses, the pharynx–cervical esophagus and cervical–upper thoracic esophagus boundaries were significantly associated with body height and age, and the upper–middle thoracic esophagus boundary was significantly associated with body height, body weight, and age (Table 1). Among these three factors, body height had the highest model coefficients and lowest *p‐*values for all boundaries. The coefficient of determination (R^2^), indicating how well a model explained the outcome, was highest for the upper–middle thoracic esophagus boundary (0.64), followed by the cervical–upper thoracic esophagus (0.48) and pharynx–cervical esophagus boundaries (0.40). The associations between body height and the distances of the three boundaries from the superior incisor dentition are shown in Figure 3.

**TABLE 1:** Multiple regression analyses of distances of boundaries from superior incisor dentition and body height, body weight, and age.

		Model coefficient	*p*‐Value	R^2^
PhCe	Body height	0.10	<0.0001	0.40
	Body weight	0.01	0.3705	
	Age	0.06	0.0009	
CeUt	Body height	0.17	<0.0001	0.48
	Body weight	−0.03	0.1301	
	Age	0.04	0.0156	
UtMt	Body height	0.20	<0.0001	0.64
	Body weight	−0.04	0.0132	
	Age	0.06	0.0001	

Abbreviations: Ce, cervical esophagus; Mt, middle thoracic esophagus; Ph, pharynx; Ut, upper thoracic esophagus.

### Evaluation of indicators

We evaluated the adequacies of pre‐existing indicators, mean distances of anatomical landmarks from the superior incisor dentition, and endoscopic landmarks, using the measured distance of the anatomical landmarks from the superior incisor dentition in each patient as the reference standard.

*Indicators based on existing landmarks*: The existing landmark of the pharynx–cervical esophagus boundary is the lower end of the palisade vessels in the cervical esophagus.^15^ The mean (SD) distance of this indicator from the superior incisor dentition was 19.8 (1.4) cm. The existing indicators of the cervical–upper thoracic esophagus and upper–middle thoracic esophagus boundaries were 18 and 24 cm from the superior incisor dentition, respectively^7^, ^13^; however, these existing indicators differed from the mean distances of the anatomical landmarks (16.9, 21.7, and 29.0 cm, respectively).

**FIGURE 4:**
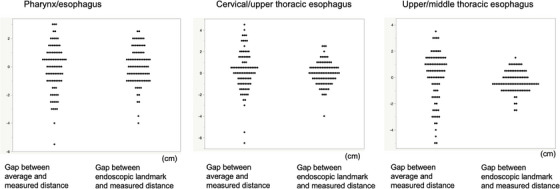
Differences between mean and measured distance, between distances based on endoscopic landmarks and measured distances, and between average and measured distances for all patients.

*Indicators based on the anatomical landmarks*: The mean distances from the superior incisor dentition to the anatomical landmarks were 16.9 cm for the pharynx–cervical esophagus, 21.7cm for the cervical–upper thoracic esophagus, and 29.0 cm for the upper–middle thoracic esophagus. These values were rounded to 17, 21.5, and 29 cm, respectively, for convenient clinical use. The differences between the mean and measured distances for all patients are shown in Figure 4. The variances in the differences were large for all three landmarks (2.8, 3.4, and 3.7, respectively).

*Indicators based on endoscopic landmarks*: The mean distances from the superior incisor dentition to the endoscopic landmarks were 16.8 cm for the pharynx–cervical esophagus, 21.7 cm for the cervical–upper thoracic esophagus, and 29.3 cm for the upper–middle thoracic esophagus, consistent with the mean distances of the anatomical landmarks (16.9, 21.7, and 29.0 cm, respectively). The differences between the distances based on endoscopic landmarks and anatomical landmarks for all patients are shown in Figure 4. The variances in the differences were 1.7, 1.2, and 0.6, respectively, which were significantly smaller than those for the indicators based on the mean distances of the anatomical landmarks. Based on these analyses of mean distance and variance, endoscopic landmarks showed the greatest consistency with the measured distances.

## DISCUSSION

We identified appropriate endoscopic landmarks for esophageal subsite classification. Individual endoscopic landmarks, such as compression of the left main bronchus, showed the best consistency with the measured distances of anatomical landmarks from the superior incisor dentition, as the reference standard in this study.

The appropriateness of endoscopic subsite classification with reference to anatomical classification has not been investigated, because determining the consistency between endoscopic and anatomical subsite classification is challenging. However, in this investigation, we compared endoscopic and anatomical subsite classification by integrating the findings of conventional endoscopy, EUS, and CT scans. Although this method is slightly complicated, we considered that it provided an accurate method for identifying suitable endoscopic landmarks for esophageal subsite classification.

Individual treatment strategies for esophageal cancers at different subsites have been determined based on anatomical subsite classification.^10^, ^11^, ^12^ The pharyngoesophageal boundary separates the pharynx and esophagus, and at the same time classifies treatment strategies for pharyngeal cancer and esophageal cancer, respectively.^5^, ^6^ Similarly, boundaries between esophageal subsites are important for classifying cancer‐treatment strategies for each subsite.^5^, ^7^, ^8^ For example, common lymph node metastatic sites differ among the cervical, upper thoracic, middle thoracic, and lower thoracic esophagus,^9^ and the extent of lymphadenectomy thus also differs depending on the esophageal subsite.^10^, ^11^ Similarly, the radiation field of CRT also depends on the esophageal subsite.^7^, ^8^, ^12^ In the case of advanced cancers that were identifiable by CT scans, their locations were also accurately diagnosed based on the anatomical landmarks, which were also identifiable by CT scans. However, for superficial cancers that were not identifiable by CT scans, their locations were diagnosed based on previous endoscopic landmarks that were not consistent with the anatomical landmarks. The endoscopic landmarks identified in this study were more consistent with anatomical landmarks than previous existing landmarks, and may thus improve the classification of esophageal subsites and contribute to the appropriate application of subsite‐specific treatment strategies.

In this investigation, we identified two indicators for esophageal subsite classification: the mean distances of anatomical landmarks from the superior incisor dentition (17 for the pharynx–cervical esophagus, 21.5 cm for the cervical–upper thoracic esophagus, and 29 cm for the upper–middle thoracic esophagus) and individual endoscopic landmarks, such as compression of the left main bronchus. The mean distances of anatomical landmarks from the superior incisor dentition are simple and easy‐to‐use landmarks; however, they vary, mainly depending on body height. Conversely, individual endoscopic landmarks provide accurate indicators of esophageal subsite classification, showing good consistency with anatomical landmarks. We thus recommend using individual endoscopic landmarks for subsite classification of lesions if the clinical management differs according to the subsite.

We investigated the boundaries between the pharynx and cervical esophagus, the cervical and upper thoracic esophagus, and the upper and middle thoracic esophagus in this study, but did not include other boundaries for which established endoscopic landmarks already exist. The boundary between the stomach and esophagus (esophagogastric junction) is determined by the lower end of the palisade vessels or the oral end of the longitudinal fold of the greater curvature, and the boundary between the middle and lower esophagus is defined as the midpoint of the tracheal bifurcation and esophagogastric junction. All subsites can thus be classified using individual endoscopic landmarks identified in this investigation or existing landmarks.

This study had some limitations. First, the sample size was small and was limited by the inclusion of patients with both EUS and chest CT data. However, the number of patients was sufficient to detect a significant difference between the two indicators, the mean distances of the anatomical landmarks from the superior incisor dentition and individual endoscopic landmarks. Another limitation was the exclusion of patients with a scar near the boundary of the esophageal subsites. We excluded these patients because we could not accurately identify endoscopic landmarks, such as compression of the left main bronchus or lower end of the palisade vessels in the cervical esophagus.

In conclusion, pre‐existing indicators of esophageal subsite boundaries are not consistent with the anatomical boundaries. Modification of the distance from the superior incisor dentition based on the average distances of the anatomical landmarks, or use of endoscopic landmarks, such as compression of the left main bronchus, is recommended to improve consistency with the anatomical boundaries.

## CONFLICT OF INTEREST STATEMENT

Ryu Ishihara has received honoraria from Olympus Corporation, FUJIFILM Medical Co., Ltd, Daiichi‐Sankyo, Miyarisan Pharmaceutical Co Ltd, AI Medical Service, AstraZeneca, and Japan Gastroenterological Endoscopy Society.

Satoki Shichijo has received honoraria from AI Medical Service Inc, EA Pharma, AstraZeneca, and Jannsen Pharmaceutical.

Takashi Kanesaka has received honoraria from Olympus Corporation and Astra Zeneca.

Yoji Takeuchi has received honoraria from Olympus Corporation, Boston Scientific Japan, FUJIFILM Medical Co., Ltd, Daiichi‐Sankyo, Miyarisan Pharmaceutical, Asuka Pharmaceutical, AstraZeneca, EA Pharma, Zeria Pharmaceutical, Kaneka Medix, Kyorin Pharmaceutical, and Japan Gastroenterological Endoscopy society.

Noriya Uedo has received honoraria from Olympus Corporation, Boston Scientific Japan, FUJIFILM Medical Co., Ltd, Daiichi‐Sankyo, Takeda Pharmaceutical, EA Pharma, Otsuka Pharmaceutical, AstraZeneca, and Miyarisan Pharmaceutical. Other authors have no financial relationships to disclose.

Yoji Takeuchi is an associate editor of DEN Open.

## Data Availability

Data is accessible by contacting the corresponding author, however, data share is not approved by the institutional review board.
